# The Effects of *Vaccinium myrtillus* Extract on Hamster Pial Microcirculation during Hypoperfusion-Reperfusion Injury

**DOI:** 10.1371/journal.pone.0150659

**Published:** 2016-04-12

**Authors:** Teresa Mastantuono, Noemy Starita, Daniela Sapio, Sabato Andrea D’Avanzo, Martina Di Maro, Espedita Muscariello, Marco Paterni, Antonio Colantuoni, Dominga Lapi

**Affiliations:** 1 Department of Clinical Medicine and Surgery, “Federico II” University Medical School, Naples, Italy; 2 CNR Institute of Clinical Physiology, Pisa, Italy; USF Health Morsani College of Medicine, UNITED STATES

## Abstract

**Introduction:**

The present study was aimed to assess the *in vivo* hamster pial microvessel alterations due to 30 min transient bilateral common carotid artery occlusion (BCCAO) and reperfusion (60 min); moreover, the neuroprotective effects of *Vaccinium myrtillus* extract, containing 34.7% of anthocyanins, were investigated.

**Materials and Methods:**

Two groups of male hamsters were used: the first fed with control diet and the other with *Vaccinium myrtillus* supplemented diet. Hamster pial microcirculation was visualized by fluorescence microscopy through an open cranial window. Pial arterioles were classified according to Strahler’s method.

**Results:**

In age-matched control diet-fed hamsters, BCCAO caused a decrease in diameter of all arterioles. At the end of reperfusion, the reduction of diameter in order 3 arterioles was by 8.4 ± 3.1%, 10.8 ± 2.3% and 12.1 ± 1.1% of baseline in the 2, 4 and 6 month control diet-fed hamsters, respectively. Microvascular permeability and leukocyte adhesion were markedly enhanced, while perfused capillary length (PCL) decreased. The response to acetylcholine and papaverine topical application was impaired; 2’-7’-dichlorofluoresceine-diacetate assay demonstrated a significant ROS production. At the end of BCCAO, in age-matched *Vaccinium myrtillus*supplemented diet-fed hamsters, the arteriolar diameter did not significantly change compared to baseline. After 60 min reperfusion, order 3 arterioles dilated by 9.3 ± 2.4%, 10.6 ± 3.1% and 11.8 ± 2.7% of baseline in the 2, 4 and 6 month *Vaccinium myrtillus* supplemented diet-fed hamsters, respectively. Microvascular leakage and leukocyte adhesion were significantly reduced in all groups according to the time-dependent treatment, when compared with the age-matched control diet-fed hamsters. Similarly, the reduction in PCL was progressively prevented. Finally, the response to acetylcholine and papaverine topical application was preserved and there was no significant increase in ROS production in all groups.

**Conclusions:**

In conclusion, *Vaccinium myrtillus*extract protected pial microcirculation during hypoperfusion-reperfusion, preventing vasoconstriction, microvascular permeability, leukocyte adhesion, reduction in PCL and preserving the endothelium function.

## Introduction

Oxidative stress has been suggested to play a key role in the pathophysiology of cerebral ischemia and reperfusion injury. Reactive oxygen species (ROS) have been implicated in brain injury after ischemia, rapidly overwhelming antioxidant defense causing further tissue damage. Moreover, the rapid restoration of blood flow increases the level of tissue oxygenation and induces a second burst of ROS generation leading to reperfusion injury by an imbalance between ROS production and antioxidant defenses. Exposure to elevated ROS concentrations causes oxidation of proteins, nucleic acids and plasma membranes. Therefore, they induce damage to blood-brain barrier, cerebral edema and inflammation [1–3], contributing to impairment of endothelial function [4].

Previous studies indicate that natural substances are able to neutralize the ROS effects in several experimental models [5]. Therefore, we tried to assess the protection exerted by antioxidant natural substances.

The present study was aimed to assess the *in vivo* hamster pial microcirculation alterations due to transient bilateral common carotid artery occlusion (BCCAO) followed by reperfusion; moreover, the protective effects of a diet supplemented with bilberry (*Vaccinum myrtillus*) were investigated.

We chose *Vaccinium myrtillus* (native of Europe and North America, a low-growing shrub of *Eriacaceae* family), because it has been found to have protective effects under various pathophysiological conditions, such as cardiovascular disorders, aging-induced oxidative stress, inflammatory responses and numerous degenerative diseases [6], [7]. However, there are no *in vivo* data about *Vaccinium myrtillus* effects on cerebrovascular alterations caused by hypoperfusion and reperfusion. This study consisted of the following steps: first, we determined the geometric characteristics of pial microvascular networks using Strahler’s method to identify the distribution of vessels, because the structural arrangements of arteriolar terminal branchings are important to modulate the blood flow to cerebral tissue [8], [9]. Successively, we evaluated the microvascular injury parameters, such as arteriolar diameter, microvascular permeability, leukocyte adhesion, capillary perfusion and the arteriolar responsiveness to acetylcholine (Ach) or papaverine (Pap) topical application. Finally, we estimated the neuronal damage by 2,3,5-triphenyltetrazolium chloride (TTC) staining and ROS production by 2’-7’-dichlorofluoresceine-diacetate (DCFH-DA) assay.

## Materials and Methods

### Experimental groups

Two hundred male hamsters (Golden hamsters, Charles River, Calco, Italy), 80–120 g body weight, were randomly divided into two groups: age-matched control diet group and age-matched *Vaccinium myrtillus* supplemented diet group.

The animals of the first group were differentiated in three sham-operated (S_1_,S_2_ andS_3_) and three hypoperfused subgroups (I_1_,I_2_ and I_3_). S_1_,S_2_ andS_3_ subgroups (n = 15 for each of these clusters) were fed with a control diet for two or four or six months, respectively, and were not submitted to BCCAO. I_1_,I_2_ and I_3_ subgroups (n = 15 for each of these clusters) were fed with a control diet for two or four or six months, respectively, and subjected to 30 min BCCAO plus 60 min reperfusion.

Similarly the animals of the second group were subdivided in three sham-operated (S_M1_, S_M2_ and S_M3_) and three hypoperfused subgroups (I_M1_, I_M2_ and I_M3_). S_M1_, S_M2_ and S_M3_ subgroups (n = 15 for each of these clusters) were fed with *Vaccinium myrtillus* supplemented diet for two or four or six months, respectively, and were not submitted to BCCAO. I_M1_, I_M2_ and I_M3_ subgroups (n = 15 for each of these clusters) were fed with *Vaccinium myrtillus* supplemented diet for two or four or six months, respectively, and subjected to 30 min BCCAO plus 60 min reperfusion.

In each subgroup six animals were used to test the pial arteriolar responses to topically applied Ach (10^−6^ M, n = 3) or Pap (10^−4^ M, n = 3) under baseline conditions and after reperfusion. Moreover, six hamsters, belonging to each subgroup, were utilized for microvascular studies, while three ones were treated with artificial cerebrospinal fluid (aCSF) containing 250 mM DCFH-DA.

To verify the effects of anesthesia on hamster microcirculation, ten animals were fed with *Vaccinium myrtillus* supplemented diet for two months, anesthetized with α-chloralose without fentanyl (I_M_C subgroup, n = 5) or pentobarbital (I_M_P subgroup, n = 5) and subjected to 30 min BCCAO plus 60 min reperfusion. These animals were compared with hamsters fed with standard diet and observed at 2 months; they were anesthetized with α-chloralose (IC subgroup, n = 5) or pentobarbital (IP subgroup, n = 5).

### Vaccinium myrtillus enriched diet

All groups were fed with 2014 Teklad Global 14% Protein Rodent Diet (Harlan, Italy), containing 48.0% carbohydrate, 14.0% protein and 4.0% fat, with energy density of 2.9 kcla/gr. For *Vaccinium myrtillus* supplemented diet, the standard diet was supplemented by adding lyophilized *Vaccinium myrtillus* (Indena S.p.A., Milan, Italy), containing 34.7% anthocyanins, expressed as cyanidin-3-glucoside: 200 mg/100g b.w./day of *Vaccinium myrtillus* extract (equivalent to 2.2 g of fresh *Vaccinium myrtillus*) were dissolved in 1 ml of distilled water and orally administered by a syringe.

### Animal preparation

All experiments conform to the *Guide for the Care and Use of Laboratory Animals* published by the US National Institutes of Health (NIH Publication No. 85–23, revised 1996) and to institutional rules for the care and handling of experimental animals. The protocol was approved by the “Federico II” University of Naples Ethical Committee (Protocol No. 3685/13/CB). All surgery was performed under α-chloralose anesthesia and all efforts were made to minimize animal suffering. Therefore, we used intramuscular (i.m.) injection of fentanyl, 2 mg/Kg b.w. (Sigma-Aldrich); moreover, a supramaximal dose of α-chloralose was administered to sacrifice the rats at the end of experiments.

Animals were individually housed in stainless steel mesh cages in a temperature-controlled (20–22°C) room with a 12:12 h light/dark cycle. They were provided with food and water *ad libitum*; food intake was measured daily by subtracting the weight of feed remaining at the end of the day. After an overnight food restriction, hamsters were anesthetized with an initial intraperitoneal (i.p.) injection of α-chloralose (50 mg/Kg b.w.) and maintained by repeated intravenous (i.v.) injections of α-chloralose (30 mg/Kg b.w. every hour). Animals were paralyzed with tubocurarine chloride (1 mg/Kg•h, i.v.), tracheotomized and mechanically ventilated with room air and supplemental oxygen. The right and left common carotid arteries were isolated for successive clamping. Then, the left femoral artery and right femoral vein were catheterized: the arterial catheter was used to measure arterial blood pressure and blood gases; the second one was utilized to administer additional anesthesia and fluorescent tracers [fluorescein isothiocyanate bound to dextran, molecular weight 70 kDa (FD 70), 50 mg/100 mg b.w., i.v. as 5% wt/vol solution in 3 min administered just once at the beginning of experiment after 30 min of the preparation stabilization; rhodamine 6G, 1 mg/100 g b.w. in 0.3 mL, as a bolus with supplemental injection throughout BCCAO and reperfusion (final volume 0.3 ml·100 g^−1^·h^−1^)]. Blood gas measurements were carried out on arterial blood samples withdrawn from arterial catheter at 30 min time period intervals. Throughout all experiments hamsters were secured on a heating stereotaxic frame to preserve the animal temperature at 37.0 ± 0.5°C. Core body temperature was monitored through a rectal probe. Moreover, mean arterial blood pressure (MABP), heart rate, respiratory CO_2_ and blood gases values were recorded and maintained stable within physiological ranges.

To observe the pial microcirculation, a cranial window (4 x 5 mm) was prepared above the left frontoparietal cortex (posterior 1.18 mm to bregma, lateral 2.10 mm to the midline), according to the method previously described [10], [11]. Briefly, a 1 cm incision was made in the skin to expose the skull and a craniotomy was performed. Cold saline solution was suffused on the skull during drilling to avoid overheating of cerebral cortex. The window inflow and outflow were assured by two needles secured in the walls of the skin, adjusted to a well, so that the dura mater was continuously superfused with artificial cerebrospinal fluid (aCSF) [12], [13]. The rate of superfusion was 0.5 mL/min controlled by a peristaltic pump. The composition of the aCSF was: 119.0 mM NaCl, 2.5 mM KCl, 1.3 mM MgSO_4_·7 H_2_O, 1.0 mM NaH_2_PO_4_, 26.2 mM NaHCO_3_, 2.5 mM CaCl_2_ and 11.0 mM glucose (equilibrated with 10.0% O_2_, 6.0% CO_2_ and 84.0% N_2_; pH 7.38 ± 0.02). The temperature was maintained at 37.0 ±0.5°C with a water bath.

BCCAO was obtained by placement of two a traumatic microvascular clips on common carotid arteries, previously isolated. After 30 min, the clamps were removed and the pial microcirculation was observed for 60 min (reperfusion). During this period microvascular responses were studied.

### Fluorescence intravital microscopy

Observations of pial microcirculation were carried out by a fluorescence microscope (Leitz Orthoplan) fitted with long-distance objectives [2.5x, numerical aperture (NA) 0.08; 10x, NA 0.20; 20x, NA 0.25; 32x, NA 0.40], a 10x eyepiece and a filter block (Ploemopak, Leitz). Epiillumination was provided by a 100 W mercury lamp using the appropriate filters for FITC, for rhodamine 6G and a heat filter (LeitzKG1). The pial microcirculation was televised with a DAGE MTI 1000 low-light level camera and recorded by a computer based frame grabber (Pinnacle DC 10 plus, Avid Technology, MA, USA).

### Geometric analysis of arteriolar networks

Under baseline conditions, the arteriolar networks were mapped by stop-frame images and pial arterioles were classified according to a centripetal ordering scheme (Strahler’s method, modified according to diameter), as previously described [9].

Pial arterioles were classified according to Strahler’s scheme: order 0 was assigned to the capillaries; thereafter, the terminal arterioles were assigned order 1 and the vessels upstream were assigned progressively higher order. When two vessels of the same order joined, the parent vessel was assigned the next higher order. If two daughter vessels were of different orders, the parent vessel retained the higher of the two orders. The procedure of the pial arteriole classification was previously reported [9]. In pial microvascular system, each blood vessel between 2 nodes of bifurcation is called a segment. In the diameter-defined Strahler model there are segments connected in series. The serially connected segments function as a single tube in hemodynamics, each tube of which is called an element. The ratio of the total number of vessel segments to the total number of vessel elements in any given order is the S/E ratio. This ratio might be a significant parameter to define symmetry (= 1) or asymmetry (>1) of microvascular branchings and, consequently, distribution of blood flow in microcirculation.

Moreover, further information may be provided by the “connectivity matrix” clarifying the number and order of daughter arterioles spreading from parent vessels. Briefly, order *n* vessels may spring from orders *n* + 1, *n* + 2, …. vessels, the component of which in row *n* and column *m* was the ratio of the total number of elements of order *n* sprung from elements of order *m*[10].

### Microvascular parameter evaluation

Microvascular measurements were made off-line using a computer-assisted imaging software system (MIP Image, CNR, Institute of Clinical Physiology, Pisa, Italy). Recording of microvascular images was performed for 1 min every 5 min during baseline, before BCCAO and at the beginning of reperfusion. Afterwards, recording was carried out every 10 min during BCCAO and the remaining reperfusion. The baseline conditions were represented by microvascular values detected within 2 min of FITC administration. In each animal, one order 4, two order 3 and two order 2 arterioles were studied during each experiment.

Arteriolar diameters were measured with a computer-assisted method (MIP Image program, frame by frame). The results of diameter measurements were in accord with those obtained by shearing method (±0.5 μm). To avoid bias due to single operator measurements, two independent “blinded” operators measured the vessel diameters. Their measurements overlapped in all cases.

The increase in permeability was calculated and reported as normalized grey levels (NGL): NGL = (I—Ir)/Ir, where Ir is the average baseline grey level at the end of vessel filling with fluorescence (average of 5 windows located outside the blood vessels with the same windows being used throughout the experimental procedure), and I is the same parameter at the end of BCCAO or at the end of reperfusion. Grey levels ranging from 0 to 255 were determined by the MIP Image program in five regions of interest (ROI) measuring 50 x 50 μm (10x objective). The same location of ROI during recordings along the microvascular networks was provided by a computer-assisted device for XY movement of the microscope table.

Adherent leukocytes (i.e., cells on vessel walls that did not move over a 30-second observation period) were quantified in terms of number/100 μm of venular length (v.l.)/30s using higher magnification (20 x and 32 x, microscope objectives). In each experimental group forty five venules were studied.

Perfused capillary length (PCL) was measured by MIP image in an area of 150 x 150 μm and was reported in μm. In this system the length of perfused capillaries is easily established by the automated process because it is outlined by dextran [14], [15].

Mean arterial blood pressure (Viggo-Spectramed P10E2 trasducer—Oxnard, CA—connected to a catheter in the femoral artery) and heart rate were monitored with a Gould Windograf recorder (model 13-6615-10S, Gould, OH, USA). Data were recorded and stored in a computer. Blood gas measurements were carried out on arterial blood samples withdrawn from arterial catheter (ABL5; Radiometer, Copenhagen, Denmark). The hematocrit was measured under baseline conditions, at the end of BCCAO and at the end of reperfusion.

### 2,3,5-triphenyltetrazolium chloride (TTC) staining

After 30 min BCCAO and 60 min reperfusion, hamsters were sacrificed and tissue damage was evaluated by TTC staining. The brains were cut into 1-mm coronal slices with a vibratome (Campden Instrument, 752 M). Sections were incubated in 2% TTC for 20 min at 37°C and in 10% formalin overnight. The colorless TTC is enzymatically reduced to a red formazan product by mitochondrial dehydrogenases in the viable area. The uncolored necrotic area site and extent in each section were evaluated by image analysis software (Image-Pro Plus) [16].

### 2’-7’-dichlorofluorescein-diacetate (DCFH-DA) assay

DCFH-DA (Sigma Chemical, St.Louis, MO, USA) was mixed with aCSF to obtain a 250 mM concentration and maintained at 37.0 ± 0.5°C. After removing the dura mater, this solution was superfused over the pial surface for 30 min at the beginning of reperfusion.

The lipophilic DCFH-DA is a stable non-fluorescent probe, able to cross the cell membrane into the intracellular space where it is hydrolyzed by intracellular esterases to non fluorescent dichlorofluorescein (DCFH). In the presence of ROS, DCFH is rapidly oxidized to its highly fluorescent analog (DCF). The remaining extra-cellular DCFH-DA was washed out with aCSF. The intensity of DCF fluorescence is proportional to the intracellular ROS level. The fluorescence intensity was determined by the use of an appropriate filter (522nm) and the evaluation of NGL, comparing the DCF fluorescence at the end of reperfusion (n = 3) with the baseline represented by pial surface just superfused by DCFH-DA [17].

### Statistical analysis

All data were expressed as mean ±standard error of mean (SEM). Data were tested for normal distribution with the Kolmogorov-Smirnov test. Parametric (Student’s t tests, ANOVA and Bonferroni post hoc test) or nonparametric tests (Wilcoxon, Mann-Whitney and Kruskal-Wallis tests) were used; nonparametric tests were applied to compare diameter and length data (median, max and min values) among experimental groups. The statistical analysis was carried out by SPSS 14.0 statistical package. Statistical significance was set at p<0.05.

## Results

### Food intake and body weight

No significant differences in daily food intake and body weight were detected among any of the diet subgroups. At the beginning the average body weight was 104.7 ± 7.6 g and 106.2 ± 4.5 g in age-matched control diet-fed hamsters and in *Vaccinium myrtillus* supplemented diet-fed ones, respectively. After two and four months there was increase in body weight; at the end of observations (6 months) the average body weight was 142.6 ± 5.2 g and 140.7 ± 7.5 g in age-matched control diet-fed hamsters and *Vaccinium myrtillus* supplemented diet-fed hamsters, respectively.

### Geometric characterization of arterial networks

Under baseline conditions hamster pial arterioles were classified according to diameter, length and branching. Capillaries were assigned order 0, the terminal arterioles were assigned order 1 (median diameter: 16.3 μm), while the upstream arterioles were assigned progressively higher orders from 2 up to 6 (median diameter: 25.0 μm, 35.7 μm, 56.3 μm, 83.8 μm and 126.5 μm, respectively) (Table 1). No differences were detected between the hamsters anesthetized with the different drugs.

**Table 1:** Diameter and length of each arteriolar order under baseline conditions.

Order	Arterioles (n)	Diameter (μm)	Length (μm)
		Median (min; max values)	
**6**	15	126.5	2330
		(115.0; 140.6)	(1679; 2980)
**5**	27	83.8	1135.5
		(81.2; 86.5)	(942.5; 1328.0)
**4**	63	56.3	1008.3
		(54.4; 58.2)	(902.9; 1113.7)
**3**	115	35.7	465
		(35.1; 36.3)	(431.8; 803.0)
**2**	160	25.0	263.8
		(24.8; 25.2)	(252.0; 276.0)
**1**	182	16.3	173
		(15.9; 16.3)	(156; 190)

The hamster networks were characterized by arcading anastomotic vessels: higher order arterioles were localized on the sides of the microvascular networks supplying blood into a reticulum of interconnecting lower order arterioles. Indeed, several artero-arteriolar anastomoses were observed.

In all microvascular networks diameter (1), length (2) and branching (3) distribution in successive orders of arterioles obeyed Horton’s law, according to the following equations:

Log10Dn = a + bn (1)

Log10Ln = a + bn (2)

Log10Nn = a + bn (3)

where a and b are two constants.

**Fig 1:**
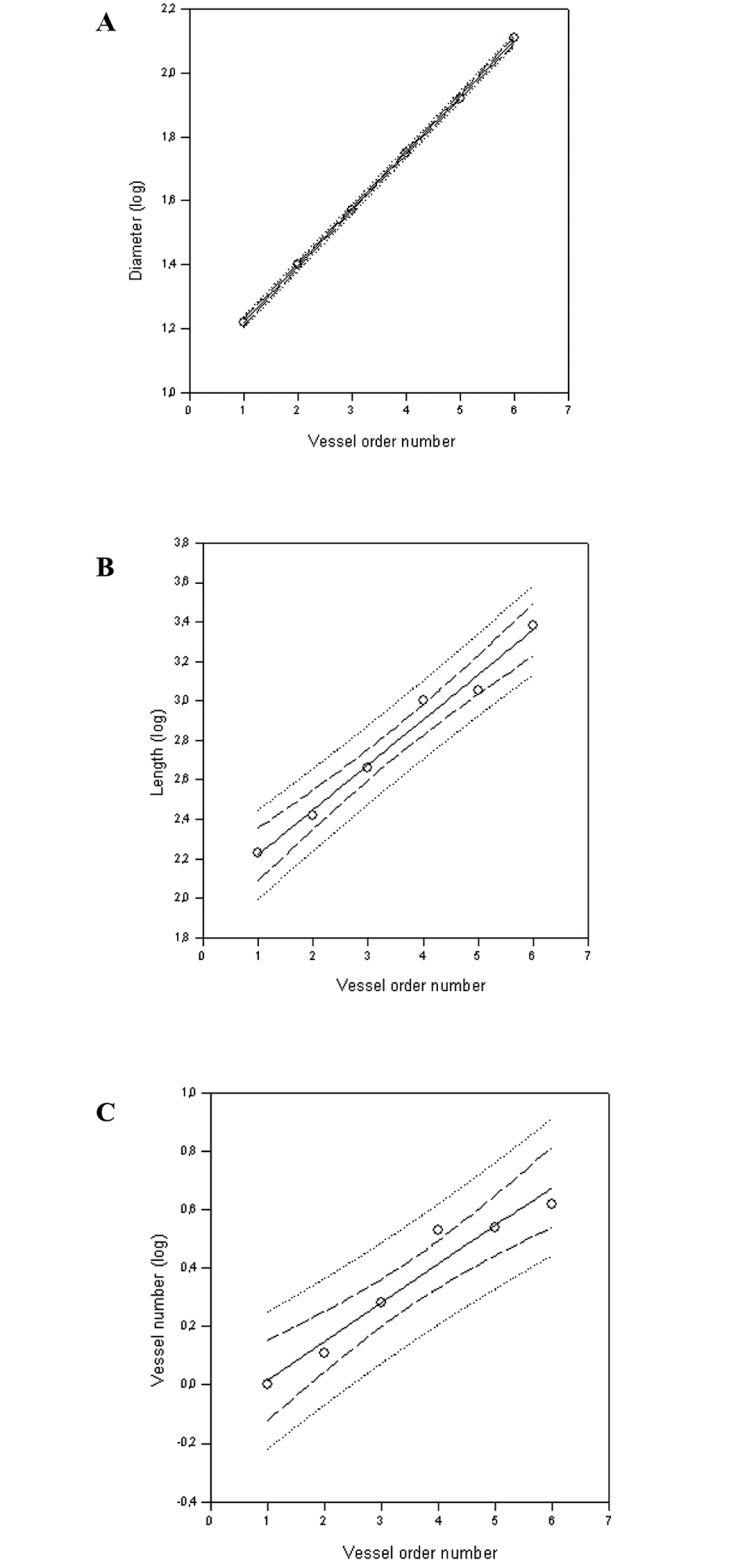
Curves obtained by Horton’s law. Relationships between mean diameter logarithm (A), length logarithm (B), logarithm of vessel element number in successive orders of vessels (C) and arteriolar order number. Increasing the number of vessel order, diameter, length and number of branching increased by a constant (fractal distribution).

The logarithm of diameter, length and branching was directly proportional to vessel order number. The ratio diameter, calculated from the slope of the curve, was 1.50 ± 0.02 and the ratio length was 1.69 ± 0.03. However, the length of order 6 vessels was not considered for this ratio calculation, because these vessels were at the borders of the cranial window. Finally, the ratio branching, dependent on vessel order and branching number, was 1.36 ± 0.05 (Fig 1), (Table 2). Therefore, the distribution of diameter, length and branching was fractal, because diameters, lengths and branchings grew as a geometric sequence with the order number. Thus, the peripheral microvascular units of each group showed a notable geometric likeness (self-similarity).

**Table 2:** Empirical constants a and b of eqs 1, 2 and 3 for semilogarithmic relationships between mean diameter, length, number of vessel elements and order number of arterioles.

*EQUATION*			
	(1) diameter	(2) length	(3) arteriolar number
	log_10_D_n_ = a + bn	log_10_L_n_ = a + bn	log_10_N_n_ = a + bn
**a**	0.177	0.228	0.133
**b**	1.043	1.992	-0.117
**R**^**2**^	1.000	0.991	0.971
**Ratio**	1.50 ± 0.02	1.69 ± 0.03	1.36 ± 0.05

A feature of the microvasculature was succession in parallel or in series of arteriolar vessels. In the pial circulation, this trend might be described by S/E ratio, which represents the total number of vessel segments divided by the total number of vessel elements in any given order. When the ratio was 1, the trend indicated complete symmetry of bifurcations; on the contrary, ratios greater than 1 denoted bifurcation asymmetry (Table 3).

**Table 3:** S/E (segments/elements ratio) in each order of vessels.

ORDER	S/E	N
**6**	4.20 ± 0.73	15
**5**	3.50 ± 0.59	27
**4**	3.40 ± 0.36	63
**3**	1.90 ± 0.12	115
**2**	1.30 ± 0.05	160
**1**	1.00 ± 0.02	182

Values are means ± SEM

N = number of vessels observed for each arteriolar order. S/E is the ratio of the total number of segments in a given order to the total number of elements in that order. This ratio is also the average number of vessel segments in series for each order of vessels

The branching vessels were described by connectivity matrix, indicating the number and order of daughter arterioles spreading from the parent vessels. In hamster pial networks, order 6 arterioles gave origin mainly to order 5 vessels [29 order 5 vessels (1.93x15)], 4 order 4 vessels (0.24x15) and 8 order 3 vessels (0.56x15) (Table 4), (Table 5). Order 5 arterioles gave origin to most order 4 [42 order 4 vessels (1.57x27)] and 3 vessels [49 order 3 vessels (1.80x27)] and few order 2 arterioles [9 order 2 vessels (0.35x27)]. Order 4 arterioles originated most order 3 [166 order 3 vessels (2.63x63)], several order 2 [45 order 2 vessels (0.72x63)] and few order 1 [11 order 1 vessels (0.18x63)]. Order 3 arterioles originated most order 2 vessels [254 order 2 vessels (2.21x115)], but also several order 3 [63 order 3 vessels (0.55x115)] and order 1 arterioles (55 order 1 vessels (0.48x115)]. Order 2 gave origin to most order 1 vessels [292 order 1 arterioles (1.83x160)]; capillaries sprung from order 2 or 1 arterioles.

**Table 4:** Connectivity matrix of pial arterioles. Values in connectivity matrix are means ± SEM. An element (m, n) in row m and column n is the ratio of the total number of elements of order m that springdirectly from parent elements of order n divided by the total number of elements of order n. The number of total vessels of order n originating from parent arterioles of order m are reported in parentheses.

***ORDER m***						
***ORDER n***						
	**1**	**2**	**3**	**4**	**5**	**6**
**0**	1.73 ± 0.52	0.27 ± 0.10	0	0	0	0
	(314)	(43)				
**1**	0.15 ± 0.06	1.83 ± 0.45	0.48 ± 0.09	0.18 ± 0.05	0	0
	(27)	(292)	(55)	(11)		
**2**	0	0.35 ± 0.13	2.21 ± 0.60	0.72 ± 0.12	0.35 ± 0.11	0
		(56)	(254)	(45)	(9)	
**3**	0	0	0.55 ± 0.12	2.63 ± 1.18	1.80 ± 0.73	0.56 ± 0.23
			(63)	(166)	(49)	(8)
**4**	0	0	0	0.14 ± 0.05	1.57 ± 0.68	0.24 ± 0.08
				(9)	(42)	(4)
**5**	0	0	0	0	0	1.93 ± 1.00 (29)
						(29)
**6**	0	0	0	0	0	0

**Table 5:** Number of vessels studied for each order of arterioles.

6	15
5	27
4	63
3	115
2	160
1	182

### Microvascular parameters

#### Sham-operated subgroups

**Fig 2:**
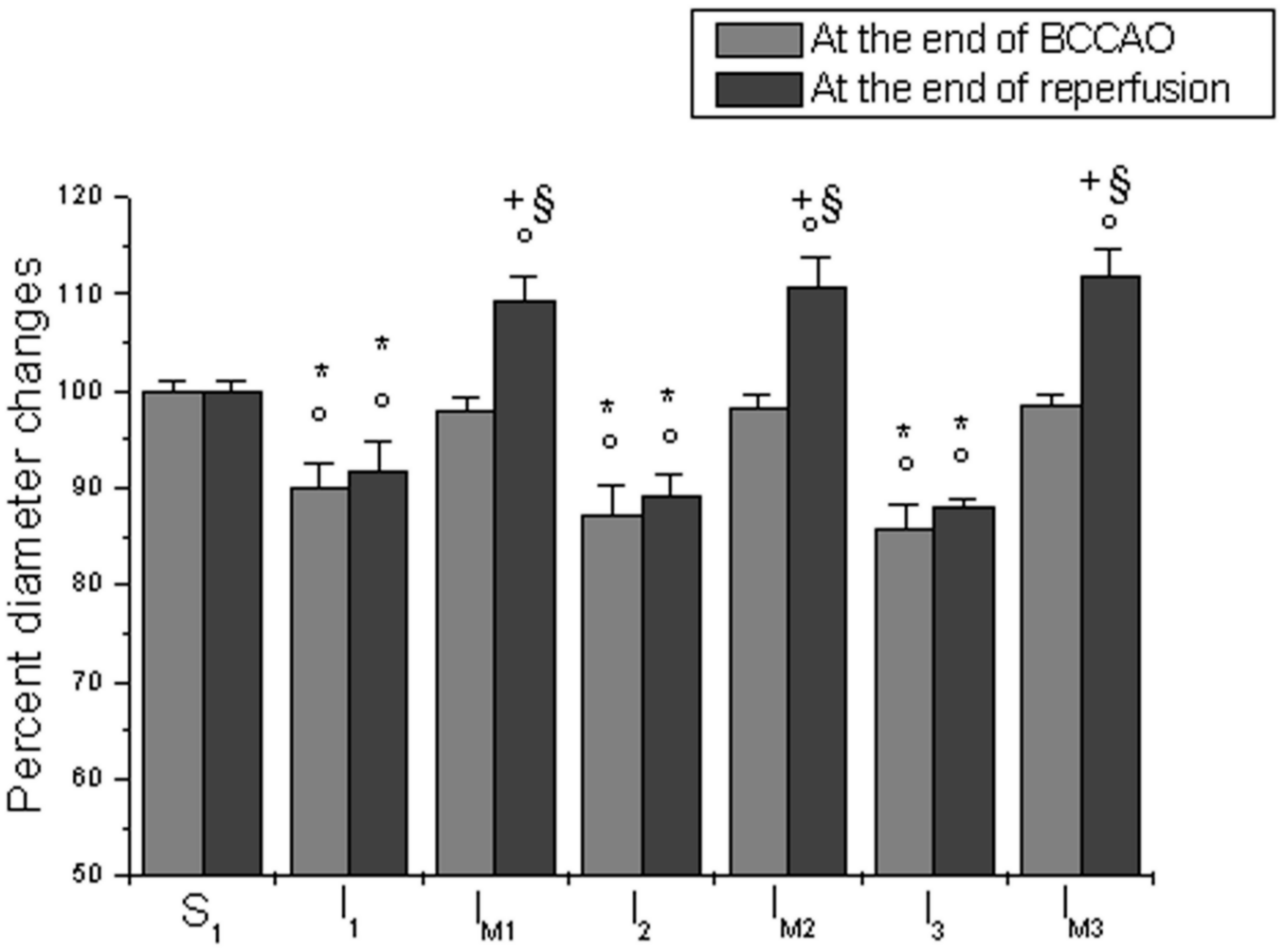
Diameter changes in the experimental groups. Diameter changes of order 3 arterioles, expressed as percent of baseline at the end of BCCAO and reperfusion, in S_1_ subgroups: and in hypoperfused subgroups (I_1_, I_M1_, I_2_, I_M2_, I_3_ and I_M3_). °p<0.01 vs. baseline; *p<0.01 vs. S_1_, S_2_, S_3_, respectively; ^+^p<0.01 vs. I_1_, I_2_ and I_3_, respectively; ^§^p<0.01 vs. S_M1_, S_M2_, S_M3_, respectively.

All sham-operated age-matched hamsters (S_1_,S_2_,S_3_,S_M1_, S_M2_ and S_M3_ subgroups) did not show significant changes in arteriolar diameter (Fig 2), microvascular permeability and leukocyte adhesion during 120 min observation; moreover, all capillaries were completely perfused (Table 6).

**Table 6:** Variations of the main parameters at the end of observation in sham-operated subgroups: S_1_, S_2_ and S_3_ hamsters fed with a control diet for two or four or six months, respectively; S_M1_, S_M2_ and S_M3_ hamsters fed with *Vaccinium myrtillus* supplemented diet for two or four or six month respectively.

Sham-operated subgroups	Microvascular leakage (NGL)	Leukocyte adhesion (Number of leukocyte/100μm of venular length/30s)	Capillary perfusion (% reduction compared to baseline)	After Ach Arteriolar diameter (%)	After Pap Arteriolar diameter (%)
	n = 6	n = 6	n = 6	n = 3	n = 3
**S**_**1**_	0.03 ± 0.01	2.0 ± 0.3	0 ± 5 vs baseline	116 ± 2 °	118 ± 4 °
			(1585 ± 45 μm)		
**S**_**2**_	0.05 ± 0.02	1.0 ± 0.5	0 ± 5 vs baseline	114.8 ± 3.0 °	117.5 ± 3.7 °
			(1570 ± 39 μm)		
**S**_**3**_	0.04± 0.02	1.0 ± 0.4	0 ± 5 vs baseline	115 ± 2 °	119 ± 3 °
			(1601 ± 50 μm)		
**S**_**M1**_	0.03 ± 0.02	2.0 ± 0.2	0 ± 5 vs baseline	113.8 ± 2.5 °	118.0 ± 3.5 °
			(1530 ± 40 μm)		
**S**_**M2**_	0.05 ± 0.01	1.0 ± 0.3	0 ± 5 vs baseline	115 ± 3 °	117.5 ± 3.4 °
			(1575 ± 49 μm)		
**S**_**M3**_	0.04 ± 0.03	1.0 ± 0.7	0 ± 5 vs baseline	114.5 ± 2.0 °	117 ± 3 °
			(1608 ± 54 μm)		

° p<0.01 vs. baseline.

Values are means ± SEM.

#### Hypoperfused subgroups

In age-matched control diet-fed hamsters (I_1_,I_2_ and I_3_ subgroups) the bilateral occlusion of common carotid arteries caused a progressive decrease in diameter of all arteriolar orders. At the end of BCCAO, order 3 arteriole diameter (n = 12 arterioles for each subgroup) was reduced by 10.0 ± 2.4%, 12.8 ±3.1% and 14.1 ± 2.3% of baseline in I_1_,I_2_ and I_3_ subgroups, respectively (p<0.01 vs. baseline and S_1_, S_2_, S_3_ subgroups) (Fig 2). Moreover, microvascular permeability (n = 30 venules for each subgroup) increased in post-capillary and connecting venules: NGL were 0.24 ± 0.01, 0.27 ± 0.02 and 0.29 ± 0.02 in I_1_,I_2_ and I_3_ subgroups, respectively (p<0.01 vs. baseline and S_1_, S_2_, S_3_ subgroups).

**Fig 3:**
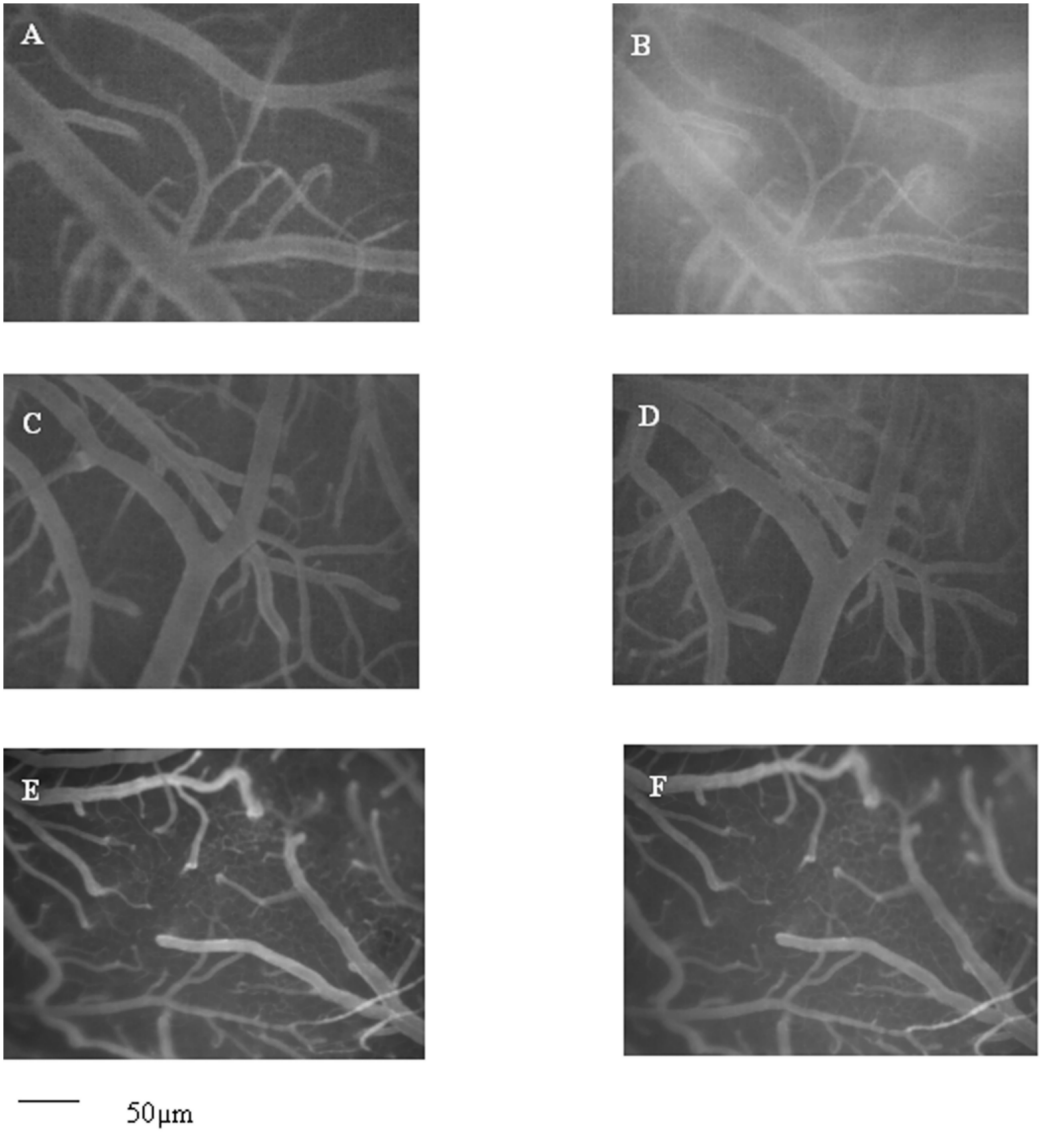
Computer-assisted images of hamster pial microvascular networks. Computer-assisted image of a pial microvascular network under baseline conditions (A) and at the end of reperfusion (B) in a hamster subjected to BCCAO and reperfusion. The increase in microvascular leakage is outlined by the marked change in the color of interstitium (from black to white). Computer-assisted images of a pial microvascular network under baseline conditions (C, E) and at the end of reperfusion in a *Vaccinium myrtillus* supplemented diet-fed hamster for four (D) and six months (F), where the leakage of fluorescent-dextran was significantly reduced. Scale bar = ___ 50μm

At the end of reperfusion, order 3 arteriole diameter (n = 12 arterioles for each subgroup) was reduced by 8.4 ± 3.1%, 10.8 ± 2.3% and 12.1 ±1.1% of baseline in I_1_,I_2_ and I_3_ subgroups, respectively (p<0.01 vs. baseline and S_1_, S_2_, S_3_ subgroups) (Fig 2). Microvascular leakage (n = 30 venules for each subgroup) was more pronounced and NGL were 0.45 ± 0.02, 0.48 ± 0.03 and 0.49 ±0.03 inI_1_,I_2_ and I_3_ subgroups, respectively (p<0.01 vs. baseline and S_1_, S_2_, S_3_ subgroups) (Fig 3). Leukocyte adhesion (n = 30 venules for each subgroup) was markedly enhanced: adherent leukocytes to venular walls were 12 ± 2, 13 ± 2 and 15 ± 3/100 μm of venular length/30 s in I_1_,I_2_ and I_3_ subgroups, respectively (p<0.01 vs. baseline and S_1_, S_2_, S_3_ subgroups). Finally, PCL significantly decreased by 45 ± 3%, 48 ± 3% and 49 ± 4% in I_1_,I_2_ and I_3_ subgroups, respectively (p<0.01 vs. baseline and S_1_, S_2_, S_3_ subgroups) (Table 7).

**Table 7:** Variations of the main parameters at the end of reperfusion in ischemic subgroups: I_1_, I_2_ and I_3_ hamsters fed with a control diet for two or four or six months, respectively and subjected to BCCAO and reperfusion; I_M1_, I_M2_ and I_M3_ hamsters fed with *Vaccinium myrtillus* supplemented diet for two or four or six month respectively, and subjected to BCCAO and reperfusion.

Hypoperfused subgroups	Microvascular leakage (NGL)	Leukocyte adhesion (Number of leukocyte/100μm of venular length/30s)	Capillary perfusion (% reduction compared to baseline)	After Ach Arteriolar diameter (%)	After Pap Arteriolar diameter (%)
	n = 6	n = 6	n = 6	n = 3	n = 3
**I**_**1**_	0.45 ± 0.02 °*	12 ± 2	45 ± 3 vs baseline	108 ± 2 *	112 ± 2
			(1597 ± 52 μm)		
**I**_**2**_	0.48 ± 0.03 °*	13 ± 2	48 ± 3 vs baseline	107 ± 2 *	110 ± 2
			(1625 ± 56 μm)		
**I**_**3**_	0.49± 0.03 °*	15 ± 3	49 ± 4 vs baseline	107 ± 3 *	111 ± 3
			(1610 ± 60 μm)		
**I**_**M1**_	0.12 ± 0.02 °^+^^§^	6 ± 3	9 ± 3 vs baseline	118 ± 3 ^+^^§^	119 ± 3 ^+^^§^
			(1630 ± 45 μm)		
**I**_**M2**_	0.09 ± 0.03 °^+^^§^	4 ± 2	7 ± 2 vs baseline	119 ± 3 ^+^^§^	118 ± 3 ^+^^§^
			(1605 ± 45 μm)		
**I**_**M3**_	0.08 ± 0.01 °^+^^§^	2 ± 1	5 ± 2 vs baseline	119 ± 2 ^+^^§^	119 ± 2 ^+^^§^
			(1620 ± 45 μm)		

°p<0.01 vs. baseline;

*p<0.01 vs. S_1_, S_2_, S_3_, respectively

^+^p<0.01 vs. I_1_, I_2_ and I_3_,respectively,

^§^p<0.01 vs. S_M1_, S_M2_, S_M3_, respectively.

Values are means ± SEM.

At the end of BCCAO, in age-matched *Vaccinium myrtillus* supplemented diet-fed hamsters (I_M1_, I_M2_ and I_M3_ subgroups) the arteriolar diameter did not significantly differ compared to baseline (Fig 2). Additionally, fluorescent spots were not detected along venular walls, indicating no increase in microvascular permeability (Table 7).

At the end of reperfusion, order 3 arteriole diameter (n = 12 arterioles for each subgroup) even increased by 9.3 ± 2.4%, 10.6 ± 3.1% and 11.8 ± 2.7% of baseline in I_M1_, I_M2_ and I_M3_ subgroups, respectively (p<0.01 vs. baseline, S_M1_, S_M2_, S_M3_ subgroups and I_1_,I_2,_ I_3_ subgroups) (Fig 2). Moreover, microvascular leakage (n = 30 venules for each subgroup) was significantly reduced compared to the previous hypoperfused subgroups: NGL were 0.12 ± 0.02, 0.09 ± 0.03 and 0.08 ± 0.01 in I_M1,_ I_M2_ and I_M3_ subgroups, respectively(p<0.05 vs. baseline, S_M1_, S_M2_, S_M3_ subgroups and I_1_,I_2_, I_3_ subgroups) (Fig 3). Similarly, leukocytes adhering to the vessel walls (n = 30 venules for each subgroup) were reduced in all treated animals: 6 ± 3, 4 ± 2 and 2 ± 1/100 μm of venular length/30 s in I_M1_, I_M2_ and I_M3_ subgroups, respectively (p<0.01 vs. I_1_,I_2,_ I_3_ subgroups). The reduction in PCL was prevented (final decrease by 9 ± 3%, 7 ± 2% and 5 ± 2% in I_M1_,I_M2_ and I_M3_ subgroups, respectively (p<0.01 vs. baseline, S_M1_, S_M2_, S_M3_ subgroups and I_1_,I_2,_ I_3_ subgroups) (Table 7).

#### IC, IP, IMC and IMP subgroups

After 30 min BCCAO, order 3 arterioles (n = 10 arterioles for each subgroup) constricted by 10.8 ± 2.8% and 11.0 ± 2.5% of baseline in IC and IP subgroups, respectively. Moreover, microvacular permeability (n = 30venules for each subgroup) was marked: NGL were 0.23 ± 0.02 and 0.26 ± 0.03 in IC and IP subgroups, respectively.

After 60 min reperfusion, the reduction in order 3 arteriole diameter(n = 10 arterioles for each subgroup) was by 8.7 ± 2.2% and 9.1 ± 2.7% of baseline in IC and IP subgroups, respectively. Additionally, NGL (n = 30venules for each subgroup) were 0.47 ± 0.03 and 0.46 ± 0.03 in IC and IP subgroups, respectively. Adherent leukocytes to venular walls (n = 30venules for each subgroup) were 11 ± 3 and 10 ± 3/100 μm of venular length/30 s in IC and IP subgroups, respectively. Finally, PCL significantly decreased by 47 ± 4% and 46 ± 3% in IC and IP subgroups, respectively.

At the end of BCCAO, no differences in arteriolar diameter and microvascular leakage were detected in hamsters belonging to I_M_C and I_M_Psubgroups, when compared to baseline.

At the end of reperfusion, order 3 arterioles (n = 10arterioles for each subgroup) dilated by 9.0 ± 1.9% and 9.8 ± 2.0% of baseline in I_M_C and I_M_P subgroups, respectively (p<0.01 vs. baseline, IC and IPsubgroups). Furthermore, NGL (n = 30venules for each subgroup) were 0.13 ± 0.02 and 0.11 ± 0.03 in I_M_C and I_M_P subgroups, respectively (p<0.05 vs. baseline, IC and IP subgroups). Leukocytes adhering to venular walls (n = 30 venules for each subgroup) were 5 ± 1 and 6 ± 1/100 μm of venular length/30 s in I_M_C and I_M_P subgroups, respectively (p<0.01 vs. IC and IPsubgroups). The reduction in PCL was slight with a final decrease by 8 ± 2%and 10 ± 2% in I_M_C and I_M_P subgroups, respectively (p<0.01 vs. baseline, IC and IP subgroups).

Finally, there were no significant changes in MABP, heart rate and hematocrit among the different groups under baseline conditions, at the end of BCCAO and at the end of reperfusion (data not shown).

### Acetylcholine and Papaverine topical application

The effects on arteriolar diameter of Ach and Pap, endothelium-dependent and -independent vasodilators, respectively, were studied. Under baseline conditions, pial arterioles markedly dilated after Ach topical application on the pial surface, but the dilation responses to Ach were impaired by BCCAO/reperfusion in I_1_, I_2_ and I_3_ subgroups. At the end of reperfusion order 3 arteriole dilation (n = 12 arterioles for each subgroup) was by 8 ± 2%, 7 ±2% and 7 ± 3% of baseline (p<0.01 vs. baseline and S_1_, S_2_, S_3_ subgroups). Conversely, the dilation was preserved in I_M1_, I_M2_ and I_M3_ subgroups: at the end of reperfusion order 3 arterioles (n = 12 arterioles for each subgroup) dilated by 18 ± 3%, 19 ± 3% and 19 ± 2% of baseline in I_M1_, I_M2_ and I_M3_ subgroups, respectively (p<0.01 vs. baseline and I_1_, I_2,_ I_3_ subgroups) (Table 7).

Under baseline conditions, topically applied Pap induced pial arteriolar dilation, that was reduced after BCCAO and reperfusion: at the end of reperfusion order 3 arterioles (n = 12 arterioles for each subgroup) dilated by 12 ± 2%, 10 ± 2% and 11 ± 3% in I_1_, I_2_ and I_3_ subgroups, respectively (p<0.01 vs. baseline and S_1_, S_2_, S_3_ subgroups). On the other hand, in the *Vaccinium myrtillus*-treated subgroups no significant differences were detected compared to the response observed in sham-operated subgroups at the end of reperfusion: order 3 arterioles (n = 12 for each subgroup) dilated by 19 ± 3%, 18 ± 3% and 19 ± 2% of baseline in I_M1_, I_M2_ and I_M3_ subgroups, respectively (p<0.01 vs. baseline and I_1_, I_2_, I_3_ subgroups) (Table 7).

### 2,3,5-triphenyltetrazolium chloride (TTC) staining

**Fig 4:**
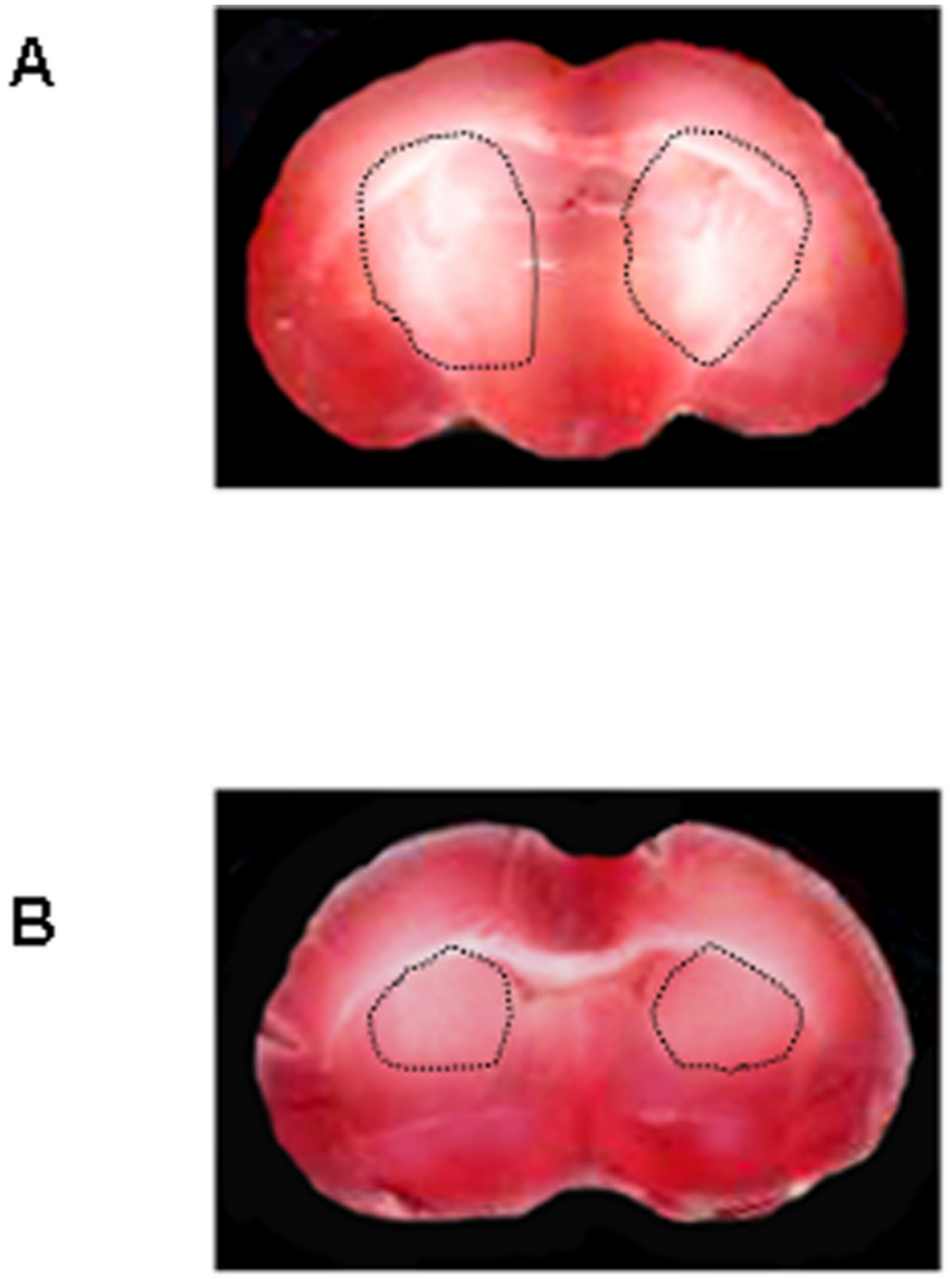
Neuronal damage. TTC staining of coronal brain slices from a hamster submitted to BCCAO and reperfusion. The lesion in the striatum is outlined by the dashed black line (A). TTC staining of coronal brain slices from a hamster treated with *Vaccinium myrtillus* supplemented diet for six months (B).

After 30 min BCCAO and 60 min reperfusion, the infarcted tissue was evident in the brain slices of age-matched control diet-fed hamsters (I_1_, I_2_ and I_3_ subgroups), mainly in the cortex and striatum. Conversely, the damaged area was drastically reduced in age-matched *Vaccinium myrtillus* supplemented diet-fed hamsters (I_M1_, I_M2_ and I_M3_ subgroups) compared to the previous subgroups (Fig 4).

### 2’-7’-dichlorofluorescein-diacetate (DCFH-DA) assay

**Fig 5:**
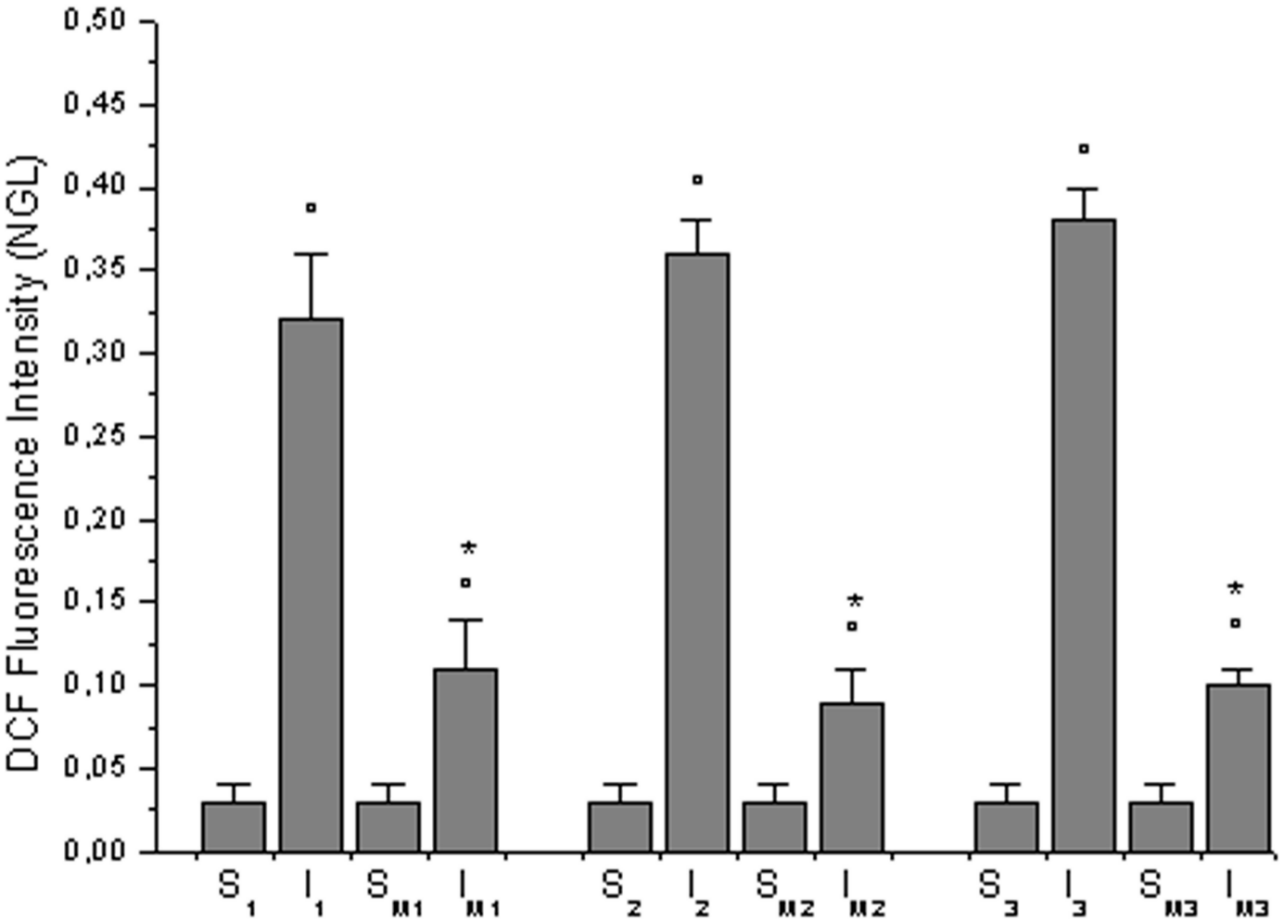
ROS formation. Changes in DCF fluorescence intensity at the end of BCCAO and reperfusion in sham-operated subgroups (S_1_, S_2_, S_3_, S_M1_, S_M2_ and S_M3_) and in hypoperfused subgroups: I_1_, I_2_, I_3_, I_M1_, I_M2_ and I_M3_. °p<0.01 vs. baseline and S_1_, S_2_, S_3_, respectively, *p<0.01 vs. S_M1_, S_M2_, S_M3_, respectively.

DCFH-DA superfusion in sham-operated rats (S_1_, S_2_, S_3_, S_M1_, S_M2_ and S_M3_ subgroups) did not cause significant increase in DCF fluorescence intensity at the end of observations (0.03 ± 0.01 NGL). In age-matched control diet-fed hamsters, submitted to BCCAO and reperfusion (I_1_,I_2_ and I_3_ subgroups, n = 3 for each subgroup), DCFH-DA superfusion induced an increase in DCF fluorescence intensity at the end of reperfusion showing an overproduction of ROS: NGL were 0.32 ± 0.04, 0.36 ± 0.02 and 0.38 ± 0.02 in I_1_, I_2_ and I_3_ subgroups, respectively (p<0.01 vs. baseline, S_1_, S_2_, S_3_ subgroups) (Fig 5).

In age-matched *Vaccinium myrtillus* supplemented diet-fed hamsters, submitted to BCCAO and reperfusion (I_M1_, I_M2_ and I_M3_ subgroups, n = 3 for each subgroup), DCF fluorescence intensity decreased compared with hypoperfused subgroups. At the end of reperfusion, NGL were: 0.11 ± 0.03, 0.09 ± 0.02 and 0.10 ± 0.01 in I_M1_, I_M2_ and I_M3_ subgroups, respectively (p<0.01 vs. baseline and I_1_,I_2_, I_3_ subgroups) (Fig 5). The pattern in DCF fluorescence intensity indicated that ROS formation was significantly reduced.

## Discussion

Our results indicate that hamster pial microvascular networks were characterized by arcading anastomotic vessels; moreover, hamster pial arterioles could be differentiated in six orders of vessels by diameter, length and branching according to Strahler’s method [8], [9]. We tried to characterize the arteriolar networks to obtain useful information on blood flow distribution in pial vessels and to evaluate the microvascular responses to hypoperfusion-reperfusion.

In hamster pial microcirculation order 6 arterioles gave origin to the arteriolar networks providing blood to successive order (from 5 to 1) vessels organized to form a reticulum of interconnected arterioles. As previously demonstrated in rat pial microvascular networks, diameter, length and branching distribution of arterioles grew with increasing vessel order; therefore, pial arteriolar networks may be defined fractal according to Horton’s law [9].

Morphometric characteristics of arteriolar networks were described in details by the connectivity matrix, useful to define the branching vessels: order 5 arterioles derived from order 6 vessels as well as order 4 vessels mainly originated from order 5 arterioles. Usually the vessel orders sprung from the immediately higher orders; capillaries originated from order 2 and 1 vessels.

Moreover, the data of the present study demonstrate that transient bilateral common carotid artery occlusion (BCCAO) and subsequent reperfusion caused a decrease in arteriolar blood flow coming from anterior vessels, while there was an increase of flow supplied by posterior arterioles. In addition, hypoperfusion and reperfusion significantly impaired vasomotor tone, blood brain barrier and tissue perfusion, consequently triggering inflammatory processes. The neuroinflammation was facilitated by leukocytes adhesion to venular vessel walls and blood brain barrier disruption, resulting in leukocyte extravasation and edema. These processes are known to trigger neuronal damage and neuronal loss. The changes in vascular tone were confirmed by altered dilation response to acetylcholine and papaverine topical application at the end of reperfusion [18].

Reactive oxygen species (ROS), produced during BCCAO and reperfusion, are known to contribute to cerebral injury. It has been reported that low antioxidant activity is related to increased cerebral lesions and neurological damage in stroke patients [19]. Our study strongly supports these findings; ROS levels, indeed, dramatically increased in the age-matched control diet-fed hamstersas shown by DCFH-DA assay at the end of reperfusion.

*Vaccinium myrtillus* supplemented diet was able to counteract microvascular impairments in hamsters according to the duration of dietary treatment. The animals, indeed, fed with supplemented diet for six months, did not show significant changes in arteriolar diameter, microvascular permeability, leukocyte adhesion and capillary perfusion during hypoperfusion and at the end of reperfusion, when there was dilation of order 3 arterioles by 11.8 ± 2.7% of baseline. Moreover, increased dilation responses to acetylcholine and papaverine were detected and accompanied by significant reduction in neuronal loss, especially in the cortex and the striatum. All these results were associated with a reduction in ROS levels in the animals fed with *Vaccinium myrtillus* supplemented diet. Therefore, it is reasonable to suggest that antioxidant properties of *Vaccinium myrtillus* extract are mainly responsible for protection of hamster pial microcirculation. In particular, in our *Vaccinium myrtillus* supplemented diet, anthocyanins (expressed as cyaniding-3-glucoside) amounted to 34.7% of extract.

To verify if fentanyl administration influenced the protective effects of *Vaccinium myrtillus* on hamster microvasculature, five animals were fed with *Vaccinium myrtillus* supplemented diet for two months and submitted to BCCAO/reperfusion after anesthesia with α-chloralose without fentanyl. Several previous studies have been carried out to investigate the vascular effects of fentanyl, but the results appear to be controversial. The effects, indeed, appear to be related to the fentanyl dosage and the experimental model utilized for the study [19–23]. In particular, in rat skeletal muscle intravenously administered fentanyl induced arteriolar dilation at higher dose and arteriolar constriction at lower dose. However, during maintenance of anesthesia arterioles constricted with higher and lower dose of fentanyl [20]. In a previous study, it has been observed that middle cerebral artery occlusion in cats caused a marked reduction in cerebral blood flow during anesthesia with pentobarbital or α-chloralose or fentanyl, while post-ischemic hyperemia was observed in cats anesthetized with pentobarbital and α-chloralose. During arterial occlusion there was no difference in cerebral blood flow in cats anesthetized with pentobarbital, α-chloralose and fentanyl [24]. The recovery of cerebral blood flow during reperfusion (120 min) was not different in these three groups of animals. Therefore, the effects induced by anesthesia with fentanyl appear to be complex and not completely clarified. However, in a previous study in golden hamsters, we did not observe significant variations in subcutaneous arteriolar blood flow during anesthesia with pentobarbital or α-chloralose, while the animals revealed resistance to the anesthesia induced by alphaxalone-alphadolone, steroid anesthetics [25]. The present data indicate that fentanyl administration at the dosage used for this study did not significantly affect the vessel diameters nor the effects exerted by *Vaccinium myrtillus* supplemented diet. The same microvascular protective effects, indeed, were detected in the animals anesthetized with α-chloralose plus fentanyl or in those treated with α-chloralose alone. A similar trend was observed in the animals anesthetized with pentobarbital. Therefore, in our experiments fentanyl administration allowed us to minimize animal suffering without interfering with the effects of *Vaccinium myrtillus* supplemented diet.

Berries (*Vaccinium spp*.) are well known as a rich source of polyphenolic compounds, most of all anthocyanins, which are secondary plant metabolites responsible for the blue, purple and red color of many plant tissues. According to Oxygen Radical Absorbance Capacity (ORAC) assay, blueberries have the highest antioxidant capacity among vegetables and fruits [26]. Several biological properties of berry anthocyanins present in berry extracts have been described, such as anti-proliferative, anti-inflammatory, antimicrobial and anti-carcinogenic activities, which are related to their potent antioxidant activity [27], [28].

Joseph et al. have studied the *in vivo* and *in vitro* antioxidant properties of berry extracts, using red blood cell resistance to ROS as a model, suggesting a positive role of blueberry dietary consumption [29]. In rodents, moreover, Joseph et al. have demonstrated that blueberry extract and blueberry supplemented diet have a tissue-protective effect under a variety of pathologic conditions, where ROS are involved. Our results support that *Vaccinium myrtillus* extract protects rat pial microcirculation blunting ROS formation with consequent tissue perfusion facilitation and blood brain barrier preservation. Moreover, the effects were evident on capillary networks, well perfused at the end of reperfusion. The capillary flow patterns can be related to arteriolar dilation likely preserved by the antioxidant properties of *Vaccinium myrtillus* extract, able to protect NO release from arteriolar endothelium, with consequent improving of tissue perfusion. The present data corroborate a previous study demonstrating that *Vaccinium myrtillus* anthocyanins (intravenously administered) are able to reduce microvascular impairments, due to ischemia-reperfusion injury in the hamster cheek pouch microcirculation [30].

Our results, moreover, indicate that *Vaccinium myrtillus* supplemented diet blunted neuronal loss as shown in TTC specimens. These data support the previous findings on neuroprotection exerted by anthocyanins. Blueberry supplemented diets, indeed, are able to slow and even reverse age-related behavioral and neuronal deficits in rodents [31–33]. In addition, Sweeny et al. have shown that blueberry supplementation protects the rat brain against neuronal damage in the hippocampus after ischemia [34], [35]. It is worth noting that bilberry extract has been shown to prevent both microvascular alterations and neuronal damage in the present hamster model of BCCAO/reperfusion.

Further studies are required to determine the polyphenols present in the *Vaccinium myrtillus* extract and their specific action mechanisms counteracting the reduction in arteriolar diameter after BCCAO and reperfusion. However, it is reasonable to conclude that the prominent effects of *Vaccinium myrtillus* extracts could be related to their natural antioxidant properties, the highest among the substances widely present in nature.
